# Quiet Quitting and Associated Factors Among Healthcare Professionals: A Systematic Review and Meta‐Analysis

**DOI:** 10.1155/jonm/2439476

**Published:** 2026-09-15

**Authors:** Ehsan Zarei, Abbas Mardani, Moretza Arab-Zozani, Aglaia Katsiroumpa, Edris Kakemam

**Affiliations:** ^1^ Department of Health Policy and Management, School of Public Health and Safety, Shahid Beheshti University of Medical Sciences, Tehran, Iran, sbmu.ac.ir; ^2^ Non-Communicable Diseases Research Center, Research Institute for Prevention of Non-Communicable Diseases, Qazvin University of Medical Sciences, Qazvin, Iran, qums.ac.ir; ^3^ Social Determinants of Health Research Center, Birjand University of Medical Sciences, Birjand, Iran, bums.ac.ir; ^4^ Clinical Epidemiology Laboratory, Faculty of Nursing, National and Kapodistrian University of Athens, Athens, Greece, uoa.gr

**Keywords:** employee engagement, healthcare professionals, job demands–resources model, nurses, quiet quitting, systematic review

## Abstract

**Background:**

Quiet quitting (QQ), the voluntary reduction of discretionary effort while maintaining minimum job requirements, has become a pressing concern in healthcare. Understanding its prevalence and drivers is essential for sustaining workforce well‐being. This systematic review and meta‐analysis aimed to estimate the pooled mean of QQ among healthcare professionals (HCPs) and identify associated factors.

**Methods:**

This systematic review was carried out according to PRISMA guidelines by searching in three international databases including Web of Science, Scopus, and PubMed from 2020 to 2026. Quantitative data were synthesized using random‐effects models. Subgroup analyses were performed based on geography, professional role, and healthcare setting. Factors associated with QQ were categorized using the expanded Job Demands–Resources (JD‐R) framework.

**Results:**

Thirty‐one studies were included in the qualitative synthesis, with 25 studies (*n* = 14,508 HCPs) included in the meta‐analysis. The pooled mean QQ score was 2.49 (95% CI: 2.37–2.61), with significant heterogeneity (*I* ^2^ = 99.2%). Subgroup analyses indicated higher QQ levels among nurses compared to other professionals and among hospital‐based staff compared to those in primary care. Factors associated with QQ were clustered into four domains: (1) job demands (e.g., burnout, excessive workload, and workplace toxicity), (2) job resources (e.g., poor leadership, lack of organizational justice, and inadequate professional support), (3) personal resources (e.g., low resilience, poor coping strategies), and (4) contextual/demographic characteristics (e.g., younger age, shorter tenure). Sensitivity analyses confirmed the robustness of these findings.

**Conclusion:**

The findings suggest that QQ is an emerging and multifactorial phenomenon among HCPs, influenced primarily by structural and relational workplace factors. However, given the substantial heterogeneity, limited geographical representation, and variability in measurement approaches across studies, the pooled findings should be interpreted cautiously.

**Implications for Nursing Management:**

Healthcare organizations should prioritize “relational” resources over extrinsic rewards. Monitoring early signs of disengagement and implementing transformational leadership models can help prevent the escalation of QQ into burnout or turnover, thereby sustaining high‐quality patient care.

## 1. Introduction

The concept of “quiet quitting” (QQ), which gained significant visibility on social media platforms, is generally defined as a behavioral pattern in which employees, without formally resigning, reduce their work engagement to minimum contractual requirements [1–4]. This phenomenon is often characterized by three elements: (1) performing only essential tasks, (2) psychological detachment from work, and (3) maintaining formal employment despite reduced discretionary effort [4]. While a unified academic definition is still evolving, some literature interprets QQ as a potential reflection of a crisis of meaning in modern work environments [5] or as an effort by individuals to establish healthy boundaries between personal and professional life [1, 2]. In the post–COVID‐19 era, reports suggest that this phenomenon may have expanded as a response to widespread burnout and chronic overwork [4, 6].

From a theoretical perspective, QQ represents a deliberate reduction in discretionary effort. Social exchange theory (SET) provides a common explanation for this behavior, suggesting that employees view work relationships as reciprocal exchanges; when they perceive an imbalance between their efforts and received rewards or appreciation, engagement may be reduced to restore a sense of fairness [2, 7]. In addition to SET, several other theoretical frameworks including the job demands–resources (JD‐R) model, conservation of resources (COR) theory, psychological contract theory, and organizational justice theory, are relevant for interpreting QQ as a potential response to high job demands, declining resources, perceived breaches of psychological contracts, or procedural and distributive injustice [8].

Evidence indicates that QQ is a global concern. Data from organizations such as Gallup suggest that a significant portion of the global workforce may be engaged in this behavior, which, by some estimates, contributes to substantial economic burdens and annual productivity losses [1, 2]. In the healthcare sector, recent studies have reported varying levels of QQ; for instance, a study in Greece found that 62% of healthcare workers exhibited characteristics of QQ [9], while Xu et al. reported that approximately 47% of primary healthcare (PHC) workers met the criteria for this phenomenon [10]. Furthermore, some reports indicate that more than 77% of nurses may be affected by QQ [11]. Given that global health systems are currently facing workforce shortages alongside increasing service demands and profound burnout [12–14], the convergence of these factors necessitates a clearer understanding of the drivers behind employee disengagement.

The antecedents of QQ are multifaceted, stemming from complex interactions among individual, organizational, and relational factors [4]. At the individual level, QQ may emerge as a coping mechanism for chronic overwork and prolonged psychological strain, often associated with diminished intrinsic and extrinsic motivation [4, 5]. Organizational factors such as perceived injustice in workload and compensation, ineffective leadership styles, inadequate organizational support, and reduced professional autonomy, are frequently cited as being associated with feelings of devaluation and detachment [1, 5, 6, 15, 16]. These findings suggest that QQ is often associated with structural and environmental deficiencies within organizations rather than being solely a reflection of individual weaknesses [1].

The consequences of QQ in clinical settings are of particular concern. At the organizational level, QQ may undermine institutional functioning by reducing employee initiative and limiting engagement in discretionary or collaborative tasks [4, 6, 17]. Furthermore, evidence suggests associations between disengagement and reduced productivity, workflow disruptions, and weakened team cohesion [17–20]. In clinical settings, these outcomes have been associated with reports of unmet patient needs, reduced quality of care, and increased safety incidents [17–20].

Despite the growing recognition of QQ, the construct remains theoretically developing. Existing studies are often fragmented and lack an integrated conceptual framework compared with well‐established constructs such as burnout [4, 16]. Understanding this phenomenon and its underlying determinants is essential for informing managerial strategies and human resource policies in high‐stakes environments [4, 5]. While some systematic reviews have explored the phenomenon, to our knowledge, this is the first meta‐analysis to synthesize existing evidence on both the pooled mean of QQ among healthcare professionals (HCPs) and its associated factors.

## 2. Aims and Questions

Therefore, the aims of this systematic review and meta‐analysis are (1) to estimate the pooled mean of QQ among HCPs and (2) to identify factors associated with this phenomenon. We sought answers to the following questions:

1. What is the pooled mean of QQ among HCPs?
2. What are the associated factors contributing to QQ in this population?

## 3. Methods

### 3.1. Study Design

This systematic review and meta‐analysis was conducted in accordance with the Preferred Reporting Items for Systematic reviews and Meta‐Analyses (PRISMA) guidelines [21]. The study protocol was registered in the PROSPERO database (ID: CRD420251178752).

### 3.2. Search Process

A comprehensive search was performed across three major databases: PubMed/MEDLINE, Scopus, and Web of Science. In addition, Google Scholar was searched to capture further relevant records beyond the core databases. The initial search covered the period from 1 January 2020, to 25 July 2025. To ensure the review was fully up‐to‐date and to capture any recently published studies, an updated search was conducted on 20 May 2026. Preliminary exploratory searches using Google Scholar and MeSH terms were conducted to refine keywords, including: “quiet quit,” “health personnel,” “healthcare provider,” “medical staff,” “nurse,^∗^” “doctor,” and “physician.” Search strategies combined these terms using Boolean operators. Supporting file Table 1 presents details of the search strategy in each database. To ensure exhaustive retrieval, reference lists of all eligible studies were screened (backward citation searching), and “snowballing” (forward citation searching) was utilized. Missing or unclear data were addressed by contacting corresponding authors.

### 3.3. Selection of Studies

We included quantitative, cross‐sectional studies published in English that examined QQ among HCPs globally. Studies were required to use standardized, validated instruments and report original quantitative data.

For the systematic review, all eligible studies were retained. However, to ensure methodological rigor in the meta‐analysis, strict inclusion criteria were applied: only studies utilizing a five‐point Likert scale (1–5) and reporting necessary summary statistics (mean and SD) were included. Studies using different response ranges (e.g., 1–4 or 1–7) or lacking summary statistics were excluded from the meta‐analysis but remained included in the qualitative synthesis of associated factors. Furthermore, to avoid potential data duplication and overestimation of effects, we assessed potential overlaps in datasets from the same regions. Studies were screened based on ethical approval codes, recruitment periods, and sample characteristics; those exhibiting clear dataset overlap were excluded from the meta‐analysis, while their qualitative findings were retained.

### 3.4. Screening and Data Extraction

All search results were imported into EndNote X21, and duplicates were removed. Two reviewers (EZ and EK) independently screened titles and abstracts, followed by a full‐text review. Disagreements were resolved through discussion or consultation with a third reviewer (AM). Data extraction was performed using a piloted Microsoft Excel form, capturing: author, publication year, setting, sample size, measurement tool, outcome data (prevalence/mean/SD), and identified predictors.

### 3.5. Quality Appraisal

Study quality was appraised independently by two reviewers (EK and EZ) using the nine‐item Newcastle–Ottawa Scale (NOS) for cross‐sectional studies [50]. The NOS evaluates studies across three domains: selection of participants (maximum five stars), comparability of groups (maximum one star), and outcome assessment (maximum three stars). The total score determines overall study quality, with classifications of high (7–9 stars), moderate (4–6 stars), or low quality (0–3 stars) [51].

### 3.6. Data Analysis

Data analysis was conducted in two distinct phases: a quantitative meta‐analysis to determine the prevalence and mean scores of QQ, and a qualitative directed content analysis to synthesize the associated factors.

Quantitative phase: For the quantitative phase, meta‐analysis was performed specifically on the mean scores of QQ using CMA (version 3) software. To account for the expected variability across global studies, a random‐effects model was employed to calculate the pooled mean. A mean score above 2.06 was considered a “quiet quitter,” i.e., a person who experienced the phenomenon of QQ [11]. Heterogeneity between studies was assessed using the Q value and *I* ^2^ statistics. To explore potential sources of heterogeneity, subgroup analyses were performed based on three predefined categorical variables. Regarding income level, countries were classified into groups based on World Bank classifications (e.g., high income, middle income). For professional roles, participants were categorized into two groups, consisting of “Nurses” and “Mixed” samples (including a combination of various healthcare roles). Furthermore, work settings were divided based on the clinical environment, distinguishing between “Hospitals” and “Mixed/Other settings” (encompassing hospitals, PHC centers, and other diverse healthcare delivery venues). Finally, to ensure the robustness of the findings, sensitivity analysis was performed using the “one‐study‐removed” approach, and publication bias was evaluated through visual inspection of funnel plots and confirmed by Egger’s regression test.

Qualitative phase: Factors associated with QQ were synthesized using the expanded JD‐R framework, which categorizes workplace and individual drivers into three distinct domains. Within this model, job demands are defined as the physical, social, or organizational conditions of work that require sustained effort and may lead to strain; job resources encompass aspects of the job that facilitate goal attainment, buffer against demands, and encourage professional development [52, 53]. The expanded JD‐R framework also integrates a third domain, personal resources, which captures individual traits and circumstances that shape how professionals respond to their work environment [54]. Factors identified in the included studies that did not fit within these three domains were grouped separately under the label “demographic and contextual factors.” We employed a directed content analysis approach [55] to perform the qualitative synthesis in three iterative phases:

1. Initial Coding: First, all identified factors from the included studies were extracted and coded.
2. Categorization: These codes were then assigned to the aforementioned theoretical domains (job demands, job resources, personal resources, and contextual factors).
3. Integration and Refinement: During this phase, we examined subcategories for conceptual overlap. Categories that represented similar underlying constructs were merged to reduce complexity and improve conceptual clarity.

This multistep process ensured that the final synthesis was grounded in the original data while remaining theoretically structured. The process culminated in a narrative summary that highlights both the frequency and the relative importance of these factors across the global healthcare landscape.

## 4. Results

### 4.1. Search Results

**FIGURE 1:**
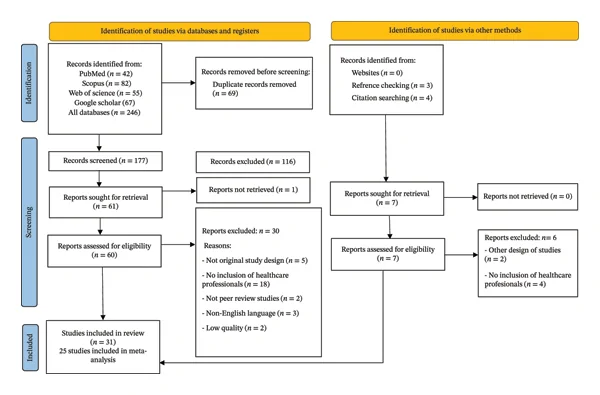
PRISMA flow diagram of selection process for identification of studies.

The initial search yielded a total of 246 records across the selected databases. After removing duplicates and conducting a multistage screening process (title/abstract and full‐text assessment), 31 studies were deemed eligible for qualitative synthesis. Of these, 25 studies met the rigorous inclusion criteria for the meta‐analysis, while 6 studies were included only in the qualitative synthesis due to inconsistent measurement scales or data overlaps. The PRISMA flow diagram (Figure 1) illustrates the comprehensive selection process.

#### 4.1.1. Study Characteristics

The included studies were published between 2024 and 2026. The geographical distribution of the included studies is as follows: Greece (*n* = 13), Turkey (*n* = 7), China (*n* = 2), Italy (*n* = 2), Egypt (*n* = 2), and one study each from Portugal, Iran, Serbia, South Korea, and Indonesia. Regarding professional roles, 21 studies focused specifically on nurses, while the remainder examined mixed populations of HCPs. In terms of work settings, 23 studies were conducted in hospital settings, with the remaining 8 studies conducted in primary care or mixed healthcare settings. Regarding measurement tools, 67.7% of the included studies (21 studies) employed a nine‐item scale to assess QQ (Table 1).

**TABLE 1:** Characteristics of included studies.

	Author/year	Country	Sample size/population	Setting	Items of QQ scale	Range of scale	Quitters (%)	QQ	Factors associated with QQ
								Mean ± SD	
1	Ventura‐Silva et al. (2026) [22]	Portugal	1097/Nurses	Hospitals and PHC centers	9 items	1–5	NR	2.5 ± 0.5	• Job burnout • Physical fatigue • Cognitive fatigue

2	Moisoglou et al. (2026) [23]	Greece	492/Nurses	Hospital	9 items	1–5	55.3%	2.18 ± 0.65	• Workplace gaslighting

3	Ghahramani et al. (2026) [24]	Iran	754/Nurses	Hospital	9 items	1–5	59.5%	2.50 ± 0.55	• Job burnout • Work setting (specialized hospitals) • Lower job satisfaction

4	Guberti et al. (2026) [25]	Italy	533/HCPs	Hospital	9 items	1–5	56.1%	2.20 ± 0.60	• Alignment between work assignment and professional request • The presence of functional and/or organizational assignments • Employment in outpatient services • Organizational mindfulness

5	El‐Sayed et al. (2026) [26]	Egypt	392/Critical care nurses	Tertiary hospitals	9 items	1–5	NR	3.53 ± 0.52	• Technostress • Low digital resilience • Higher education level • Working hours (≤ 8 h) • Work experience

6	Belli and Erçelik (2026) [27]	Turkey	400/Pediatric nurses	Hospital	7 items	7–35 (1–5)	NR	20.35 ± 4.84	• Low moral resilience

7	Bilgiç et al. (2026) [28]	Turkey	209/Midwifery and Nursing Academics	Public and private universities	34 items	38–166 (1–5)	NR	98.19 ± 21.34	• Mobbing exposure

8	Boy et al. (2026) [29]	Turkey	242/HCPs	Healthcare institutions	20 items	20–80 (1–4)	29.4%	59.33 ± 11.83	• Unfavorable working conditions • Increased workload • Low wages • Ministry policies • Communication problems • Physical and verbal violence from the patient/patient relatives • Psychological violence from team members/managers • Managers do not value employees’ opinions • Disturbed work–life balance • Insufficient use/restriction of annual leave • Lack of appreciation

9	Hu et al. (2025) [30]	China	520/PHC professionals	PHC	6 items	1–5	NR	2.12 ± 0.60	• Goal specificity and identity • Goal fulfillment and organizational support • Goal difficulty • Goal attainability • Goal conflict • Poor health status • Job position (clinical roles)

10	Danacı et al. (2025) [31]	Turkey	225/Nurses	University hospital	9 items	1–5	NR	2.48 ± 0.65	• Stress of conscience • Compassion fatigue

11	Xu et al. (2025) [10]	China	520/PHC workers	PHC	9 items	1–5	47	2.12 ± 0.61	• Poor temporal leadership • Poor time management • Low work–family enrichment

12	Toska et al. (2025) [9]	Greece	186/HCPs	Hospital	9 items	1–5	62	2.39 ± 0.59	• Conflicts with an administration employee • Conflicts with others • Promotions and career advancements • Unawareness of hospital management of staff contribution

13	Stankovic and Slavkovic (2025) [32]	Serbia	647/HCPs	Public health organizations	8 items	1–5	NR	2.45 ± 0.7	• Quality of work life • Gender (Female)

14	Rinaldi and Pomarolli (2025) [33]	Italy	76/Nurses	Hospital	9 items	1–5	46.1	2.07 ± 0.67	• Younger age • Less work experience

15	Othman et al. (2025) [34]	Egypt	482/Nurses	Hospital	7 items	1–5	NR	3.05 ± 0.99	• Perceived quiet firing

16	Moisoglou et al. (2025a) [35]	Greece	614/Nurses	Hospital	9 items	1–5	66.6	NR	• Poor engaging leadership • Younger age • Less work experience • Understaffed ward • Shift work

17	Moisoglou et al. (2025b) [36]	Greece	462/Nurses	Hospital	9 items	1–5	NR	2.42 ± 0.71	• Workplace gaslighting (Abuse of power) • Lack of resilience

18	Moisoglou et al. (2025c) [37]	Greece	425/Nurses	Hospital	9 items	1–5	66.1	2.40 ± 0.73	• Low nurse participation in hospital affairs • Poor nurse manager ability, leadership, and support • Poor collegial nurse–physician relationships • Poor nursing foundations for quality of care • Gender (male) • Working in understaffed wards • Younger age

19	Kang et al. (2025) [38]	South Korea	323/Nurses	Hospital	9 items	1–5	NR	2.58 ± 0.51	• Low organizational justice • Low job satisfaction • High role ambiguity • Infection control fatigue • Lower education level • Lower incomes • Younger age • Lower work experience • General hospital (vs. tertiary hospitals)

20	Gün et al. (2025a) [39]	Turkey	317/Nurses	Hospital	9 items	1–5	62.5	2.31 ± 0.51	• Job burnout • Turnover Intention

21	Gün et al. (2025b) [40]	Turkey	383/Nurses	Hospital	8 items	1–5	60.9	2.62 ± 0.93	• Job burnout • Low organizational Support

22	Galanis et al. (2025a) [41]	Greece	1092/Nurses	Hospital	9 items	1–5	74.3	2.60 ± 0.70	• High workloads • Job burnout

23	Galanis et al. (2025b) [42]	Greece	946/Nurses	Hospital	9 items	1–5	NR	2.36 ± 0.66	• Low job satisfaction • Job burnout

24	Fathulaela et al. (2025) [43]	Indonesia	191/Hospital employees	Hospital	17 items	1–5	NR	NR	• Poor work–life balance • Job burnout

25	Ardıç and Erişen (2025) [44]	Turkey	224/HCPs	Mixed	25 items	1–7	NR	3.79 ± 0.84 (2.86 ± 0.56)	• Work stress • Job satisfaction • Low work engagement

26	Moisoglou et al. (2024a) [45]	Greece	909/Nurses	Hospital	9 items	1–5	68.2	2.37 ± 0.67	• Gender (male) • Shift work • Understaffed workplace • Less work experience

27	Moisoglou et al. (2024b) [46]	Greece	328/Nurses	Hospital	9 items	1–5	66.2	2.39 ± 0.70	• Poor managerial support • Poor cultural support • Poor innovation support

28	Galanis et al. (2024a) [47]	Greece	957/Nurses	Hospital	9 items	1–5	71.9	2.43 ± 0.67	• Low moral resilience • Low personal integrity • Low relational integrity

29	Galanis et al. (2024b) [11]	Greece	665/Nurses	Hospital	9 items	1–5	77.3	2.5 ± 0.6	• Workplace bullying • Positive coping strategies • Negative coping strategies • Gender (Male)

30	Galanis et al. (2024c) [48]	Greece	1760/HCPs	Mixed	9 items	1–5	57.8	2.22 ± 0.66	• Nurse (vs physician and others) • Shift work • Less work experience • Job burnout • Low job satisfaction

31	Galanis et al. (2024d) [49]	Greece	992/Nurses	Hospital	9 items	1–5	74.4	2.55 ± 0.74	• Low emotional intelligence

*Note:* PHC: primary healthcare; HCPs: healthcare professionals.

Abbreviations: NR, not reported; QQ, quiet quitting.

### 4.2. Quality of Included Studies

The quality of the included studies was appraised using the NOS. The assessment revealed that 19 studies (61.3%) scored between 7 and 9 (high quality), whereas 12 articles (38.7%) achieved scores between 4 and 6, indicating moderate quality. Two studies were excluded based on low quality (0–3 stars) during the appraisal process. Detailed NOS quality assessment scores for individual studies included in the systematic review and meta‐analysis are presented in Supporting Table 2. These findings suggest a generally acceptable level of methodological quality across the synthesized body of literature.

### 4.3. Meta‐Analysis Results

**FIGURE 2:**
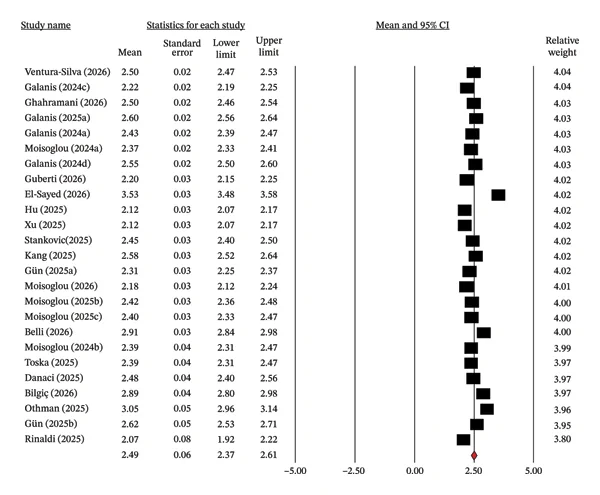
The forest plot.

A total of 25 studies, encompassing a combined sample size of 14,508 HCPs, met the inclusion criteria for the meta‐analysis of mean QQ scores. Given the substantial statistical heterogeneity observed across studies, a random‐effects model was employed to calculate the pooled estimate. The meta‐analysis revealed a pooled mean QQ score of 2.49 (95% CI: 2.37–2.61; *Z* = 41.40, *p* < 0.001). The heterogeneity metrics showed significant variation across included studies (Q = 3031.65, df = 24, *p* < 0.001; *I* ^2^ = 99.2%; *τ* ^2^ = 0.089), with a 95% prediction interval ranging from 1.83 to 3.15. The forest plot summarizing these findings is presented in Figure 2.

To assess the stability of our findings, a leave‐one‐out sensitivity analysis was performed. The results confirmed the robustness of the estimates, with pooled means ranging narrowly from 2.45 to 2.51 after excluding individual studies, indicating that no single study disproportionately influenced the overall pooled estimate. Given the high proportion of studies conducted in Greece, an additional sensitivity analysis was performed by excluding all studies from this country to assess the potential influence of regional homogeneity on the pooled estimate. Following the exclusion of 10 Greek studies, the meta‐analysis of the remaining 15 studies yielded a pooled mean of 2.56 (95% CI: 2.36–2.75). The consistency of this estimate with the overall pooled mean (2.49) confirms that the findings are robust and not disproportionately driven by the data from a single country.

**FIGURE 3:**
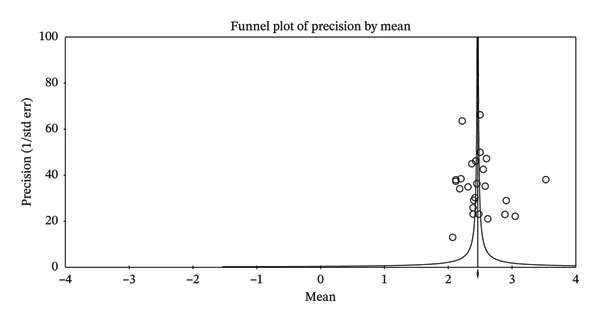
The funnel plot.

Finally, publication bias was assessed using Egger’s regression test. The intercept was 5.28 (SE = 6.85, *p* = 0.449), which was not statistically significant, suggesting no evidence of publication bias in the current meta‐analytic dataset. Nevertheless, given the substantial heterogeneity (*I* ^2^ = 99.2%), these results should be interpreted with caution. The funnel plot is presented in Figure 3.

#### 4.3.1. Subgroup Analyses

Subgroup analyses were conducted to explore potential sources of heterogeneity. The results are summarized in Table 2.

**TABLE 2:** Subgroup analysis results.

Subgroup	Categories	Frequency	Pooled mean (95% CI)	*p* value
Geographic region	Africa	2	3.29 (2.82–3.76)	< 0.001
	Asia	9	2.50 (2.32–2.68)	
	Europe	14	2.37 (2.30–2.45)	

Income level	Low income	11	2.63 (2.36–2.91)	0.084
	High income	14	2.38 (2.30–2.46)	

Profession	Nurses	19	2.57 (2.43–2.70)	< 0.001
	Other HCPs	6	2.25 (2.15–2.35)	

Healthcare setting	Hospital	20	2.53 (2.39–2.66)	0.021
	PHC	3	2.23 (2.02–2.44)	

*Note:* Two studies conducted in academic and mixed‐setting contexts were not included in the healthcare‐setting subgroup analysis, as their settings did not align with the “Hospital” or “PHC” categories used for this stratification. HCPs: healthcare professionals; PHC: primary healthcare.

Geographic region: Significant differences were observed across regions (*p* < 0.001). The highest pooled mean was in African studies (3.29; 95% CI: 2.82–3.76), followed by Asian (2.50; 95% CI: 2.322.68) and Europe (2.37; 95% CI: 2.30–2.45).

Income level: HCPs from low‐ and middle‐income countries reported higher mean QQ scores (2.63; 95% CI: 2.36–2.91) compared to high‐income countries (2.38; 95% CI: 2.30–2.46); however, the difference was not statistically significant (*p* = 0.084).

Professional Role: Nurses demonstrated significantly higher QQ scores (2.57; 95% CI: 2.43–2.70) compared to other HCPs (2.25; 95% CI: 2.15–2.35; *p* < 0.001).

Healthcare setting: Professionals working in hospital environments reported significantly higher QQ scores (2.53; 95% CI: 2.39–2.66) compared to primary care workers (2.23; 95% CI: 2.02–2.44; *p* = 0.009). Two studies conducted in academic and mixed healthcare settings were excluded from the healthcare‐setting subgroup analysis, as they did not fit within the predefined “Hospital” or “PHC” categories.

### 4.4. Factors Associated With QQ

Factors associated with QQ among HCPs were synthesized into four thematic dimensions: job demands, job resources, personal resources, and sociodemographic/contextual factors (Table 3).

**TABLE 3:** Factors associated with quiet quitting among healthcare professionals.

Category	Subcategory	Key factors	Times cited in studies
Job Demands	Psychological & Emotional	Burnout, Compassion Fatigue, Stress of Conscience, Physical/Cognitive Fatigue	12
	Workload & Structural	High Workload, Shift Work, Understaffing, Ministry Policies, Goal Conflict/Difficulty	11
	Toxic Organizational	Bullying, Mobbing, Gaslighting, Violence, Conflicts, Communication Barriers	9
	Technological	Technostress, Infection Control Fatigue	2

Job Resources	Financial & Recognition	Wages, Appreciation, Career Growth, Management Awareness	5
	Leadership & Support	Managerial Support, Quality Leadership	6
	Climate & Design	Satisfaction, Justice, Work–Life Balance, Mindfulness, Task Alignment, Innovation/Cultural Support	19

Personal Resources	Psychological Capabilities	Engagement, Integrity, Resilience, Emotional Intelligence, Time Management, Coping, Personal Health	10

Contextual/Demographic	Professional	Tenure, Job Position, Work Setting	11
	Individual	Age, Gender, Education	10

#### 4.4.1. Job Demands

Job demands consistently emerged as significant correlates of QQ. Psychological and emotional strains, including burnout, compassion fatigue, and cognitive fatigue, showed a strong positive association with QQ [22, 24, 31, 39–44, 48]. Structural demands, such as heavy workloads, shift work, and severe understaffing, were also linked to increased employee detachment [26, 29, 35, 37, 41, 45, 48]. Furthermore, external systemic pressures, such as restrictive policies and goal conflicts between clinical targets and available resources, significantly contributed to this phenomenon [29, 30]. Organizational toxicity including workplace bullying, violence, gaslighting, and interpersonal communication deficits was identified as a key driver [9, 11, 23, 28, 29, 34, 35, 37]. Finally, technological pressures, including technostress and infection‐control fatigue, were also associated with higher instances of QQ [26, 38].

#### 4.4.2. Job Resources

A significant negative association was observed between job resources and QQ, suggesting that adequate support buffers against detachment. Financial and recognition‐based deficits, such as low wages, lack of appreciation, and limited career advancement, were significantly linked to higher QQ [9, 29, 38]. Deficits in leadership and organizational support, particularly poor supervisory styles, were strongly associated with employee disengagement [10, 29, 35, 40, 46].

Regarding organizational climate and job design, factors such as low job satisfaction [24, 38, 42, 44, 48], low perceived organizational justice [38], role ambiguity [38], and work–life imbalance (e.g., restricted annual leave) were significant correlates [10, 29, 43]. In addition, lack of organizational mindfulness, poor task alignment [25], limited nurse participation in decision‐making, and insufficient institutional foundations for quality care were identified as structural deficits [37, 46].

#### 4.4.3. Personal Resources

Personal resources exhibited a consistent inverse relationship with QQ levels. Deficits in psychological and moral capabilities were linked to higher QQ, specifically low work engagement [44], low emotional intelligence [49], reduced resilience [11, 27, 35], and compromised personal or relational integrity [47]. Furthermore, cognitive‐behavioral variables, including poor time management skills [10], maladaptive coping strategies [11], and poor overall health status [30] were significantly associated with increased QQ.

#### 4.4.4. Contextual and Demographic Factors

Several contextual and demographic variables were associated with QQ. Younger age [33, 35, 37, 38], male gender [11, 37, 45], specific education levels [26, 38], and shorter organizational tenure [26, 33, 35, 38, 45, 48] were identified as significant contributors. In addition, professional roles (with nurses showing distinct patterns compared to other staff [48]), and work‐setting characteristics (e.g., outpatient versus inpatient environments) were significantly related to varying levels of QQ [24, 25, 38].

## 5. Discussion

The present systematic review and meta‐analysis aimed to estimate and highlight the mean of QQ among HCPs and identified associated factors of QQ. The results of the meta‐analysis revealed a pooled mean QQ score of 2.49 out of 5, indicating that HCPs’ perceptions of QQ are generally moderate to high. These findings suggest that a significant portion of the workforce may be reducing their discretionary effort. This aligns with broader global observations regarding workplace disengagement [1]. However, given the substantial statistical heterogeneity observed across the included studies (*I* ^2^ = 99.2%), this pooled estimate should be interpreted with caution and considered as an average across diverse contexts rather than a fixed universal rate.

Our subgroup analyses reveal that this phenomenon is not uniform; rather, it varies significantly based on professional role, work setting, and geography. Nurses exhibited higher levels of QQ compared to other professional groups. This may be attributed to the unique demands of the nursing profession, which often involve intensive patient care and high levels of emotional labor [56–58]. In environments where organizational support is insufficient, these demands may lead to higher disengagement [59], making QQ a more common coping strategy among nursing staff. Similarly, the higher prevalence of QQ in hospital settings, compared to primary care, appears to reflect the greater patient acuity and workload intensity characteristic of acute care environments [60–62]. These variations suggest that the manifestation of QQ is closely tied to the structural and operational pressures inherent in specific healthcare environments globally. However, the subgroup findings for Africa (*n* = 2) and primary care settings (*n* = 3) should be interpreted with particular caution due to the limited number of studies available for these strata.

Geographic variation was particularly notable, with significant differences observed across regions. Higher pooled QQ scores in African and Asian studies compared with European studies may reflect variations in healthcare system resources, workforce shortages, occupational stressors, organizational support structures, and cultural attitudes toward work across settings [63–65]. In addition, HCPs from low‐ and middle‐income countries demonstrated relatively higher QQ scores than those from high‐income countries, although this difference did not reach statistical significance. These patterns may indicate the influence of broader structural and socioeconomic pressures, including staffing limitations, resource constraints, and challenging working conditions in lower‐resource healthcare systems [66, 67]. Nevertheless, these findings should be interpreted cautiously given the unequal geographical distribution of studies and the limited number of studies from some regions. Importantly, sensitivity analysis excluding studies from Greece confirmed that, although the concentration of research in specific countries such as Greece and Turkey was high, the overall pooled estimate remained relatively stable, suggesting that the observed phenomenon was not merely an artifact of regional reporting.

The synthesized evidence further suggests that QQ is associated with a complex interaction between workplace demands and available resources. Interpreted through the lens of the JD‐R framework, the findings indicate that environments characterized by chronically high job demands and insufficient organizational resources may be linked with emotional exhaustion and disengagement among HCPs [40, 41, 44]. In particular, excessive workload, burnout, and workplace stressors were consistently associated with higher QQ levels both in this review and in previous research [41]. However, because all included studies were cross‐sectional, these relationships should be interpreted as associative rather than causal.

Our synthesis indicates that high job demands are the most frequently cited correlates of QQ. Factors related to workload, toxic work environments, and systemic stressors appear to be primary drivers of disengagement. Specifically, reports of burnout, excessive workload, and workplace toxicity (e.g., bullying, gaslighting) were highly prominent in the literature. These findings are consistent with the COR theory, suggesting that when professional demands chronically deplete an individual’s psychological energy, disengagement may function as a protective mechanism to prevent further strain [1, 4, 17].

Interestingly, while factors such as leadership and organizational design (e.g., team alignment, organizational mindfulness) were frequently identified (appearing in 19 studies), markers of financial compensation and extrinsic rewards were mentioned less frequently (five studies). This suggests that for many HCPs, the quality of the organizational climate, supportive leadership, and the ability to perform meaningful work may be as critical—if not more so—than immediate financial incentives. Deficits in these resources appear to break the “social contract” between the employee and the organization, leading to a reduction in discretionary effort as a means of restoring perceived equity [1, 4, 68].

Personal capabilities, such as emotional intelligence and resilience, appear to serve as buffers against QQ. Individuals with stronger internal resources seem better equipped to navigate high‐demand environments without resorting to disengagement [1, 4, 47]. Furthermore, demographic factors such as younger age and shorter tenure were associated with higher vulnerability to QQ, which may reflect a developing capacity for managing chronic occupational stress [32, 45]. Some studies suggest that men may be more prone to QQ, potentially due to weaker professional identity or a lower sense of belonging in predominantly female professions such as nursing [19, 68].

### 5.1. Limitations

Several limitations should be noted. First, the cross‐sectional nature of most included studies limits the ability to infer causal pathways; therefore, longitudinal studies are needed to better understand the temporal dynamics of QQ. Second, while we observed high statistical heterogeneity, this is common in meta‐analyses of behavioral constructs in healthcare and was addressed through prediction intervals, subgroup analyses, and sensitivity analyses. Nevertheless, the pooled estimate should be interpreted with caution. Third, the included studies used heterogeneous statistical metrics (e.g., correlation coefficients, odds ratios, and regression coefficients) to report associations with QQ, and one study reported factors based on participant‐generated responses without statistical effect sizes. Therefore, a quantitative meta‐analysis of effect sizes was not feasible, and our synthesis remains narrative. Fourth, the geographic concentration of studies, particularly from Greece and Turkey, requires cautious interpretation. Our sensitivity analysis excluding Greek studies confirmed robustness; however, the scarcity of studies from North America, Western Europe, and Australia limits global generalizability. Finally, only English‐language peer‐reviewed studies were included, while gray literature, dissertations, and conference proceedings were excluded. Given the emerging nature of QQ research, potentially relevant evidence may therefore have been missed, increasing the possibility of selection and publication bias.

### 5.2. Implications for Practical, Policy, and Research

Since structural and relational deficits are consistently associated with of QQ, interventions must prioritize workplace systemic reform:

• Structural Optimization: Implement flexible staffing models aligned with patient acuity and conduct regular workload audits to eliminate nonessential administrative tasks that drive cognitive fatigue.
• Leadership Transformation: Transition from hierarchical management to transformational leadership. Prioritize empathy and team cohesion, and establish anonymous reporting systems to enforce zero‐tolerance policies against workplace toxicity.
• Targeted Support: Launch formal mentorship programs to mitigate “reality shock” among younger staff and those with shorter tenure.
• Resilience Building: Provide evidence‐based workshops on stress management and soft skills as a supplementary support mechanism, distinct from and secondary to structural organizational changes.

Future research should shift toward longitudinal designs to clarify the progression of QQ and its impact on clinical outcomes. In addition, there is a need for standardized measurement tools to ensure consistency across studies, as the current conceptual development of QQ remains in its early stages.

## 6. Conclusion

This systematic review and meta‐analysis suggests that QQ is an emerging and multifactorial phenomenon among HCPs, particularly among nurses and hospital‐based staff. The findings indicate that QQ may be shaped by a complex interplay of job demands, job resources, personal resources, and demographic and contextual characteristics. High job demands—such as burnout, workload, role ambiguity, work–family imbalance, and workplace bullying—together with insufficient organizational support, poor leadership, and reduced quality of work life, appear to contribute to disengagement among healthcare workers. Personal characteristics, including emotional intelligence, resilience, and coping strategies, may further influence vulnerability to QQ. However, these findings should be interpreted cautiously due to the substantial heterogeneity across studies, the concentration of evidence in a limited number of countries, and the lack of standardized measurement tools for QQ. While the current evidence highlights QQ as a potentially important organizational concern with implications for workforce sustainability and patient care, further longitudinal and internationally diverse studies are needed to establish the consistency, magnitude, and consequences of this phenomenon across healthcare systems. Nevertheless, the findings support the potential value of multilevel organizational strategies aimed at reducing excessive job demands, strengthening workplace resources, and promoting supportive leadership and healthy work environments.

## Author Contributions

E.K. and E.Z. developed the design of this study. E.K. and E.Z. searched various sources by designing the search strategy. E.K., E.Z., and A.M. participated in the selection of the study, data extraction, and quality assessment. E.K. and M.A‐Z. performed the analysis of the included studies. E.K., E.Z., M.A‐Z., A.M., and A.K. drafted the first draft of the manuscript. E.Z. and A.K. revised the draft. Finally, all authors checked and approved the revised draft of the manuscript.

## Funding

No funding was received for this study.

## Conflicts of Interest

The authors declare no conflicts of interest.

## Supporting Information

Additional supporting information can be found online in the Supporting Information section.

## Supporting information


**Supporting Information 1** Table 1. Search strategy. Table 2. Risk of bias assessment of the studies based on the modified Newcastle–Ottawa tool for cross‐sectional studies.

**Supporting Information 2** PRISMA checklist.

## Data Availability

All data generated or analyzed during this study are included in this published article.
