# Supplementary material for: Quiet Quitting and Associated Factors Among Healthcare Professionals: A Systematic Review and Meta‐Analysis
Source: J Nurs Manag. 2026 Sep 15;2026:2439476. doi: 10.1155/jonm/2439476 (PMC13578937; doi:10.1155/jonm/2439476)
Supplement: Supplementary file 2 [file JONM-2026-2439476-s002.docx]

**Table 1. Search strategy**

| **Databases** | **STRATEGY** |
| --- | --- |
| PubMed | (((((((((((((((Healthcare Worker[Title/Abstract]) OR (Health Worker[Title/Abstract])) OR (Healthcare Personnel[Title/Abstract])) OR (Health Personnel[Title/Abstract])) OR (Health Manpower[Title/Abstract])) OR (Healthcare Provider[Title/Abstract])) OR (Allied Health Personnel[Title/Abstract])) OR (Medical Staff[Title/Abstract])) OR (Health Staff[Title/Abstract])) OR (health professional[Title/Abstract])) OR (healthcare professional[Title/Abstract])) OR (Nurse*[Title/Abstract])) OR (Doctor[Title/Abstract])) OR (physician[Title/Abstract])) OR (paramedic [Title/Abstract])) AND (quiet quit*[Title/Abstract]) |
| Scopus | TITLE-ABS-KEY (( quiet quit*) AND (“healthcare worker" OR "health worker" OR "healthcare personnel" OR "health personnel" OR "healthcare provider" OR "medical staff" OR "nurse*" OR "physician" OR "doctor" OR "paramedic”)) |
| Web of Sciences | TS= (quiet quit*)AND TS= ("healthcare worker" OR "health worker" OR "healthcare personnel" OR "health personnel" OR "healthcare provider" OR "medical staff" OR "health professional" OR "nurse*" OR "doctor" OR "physician" OR "paramedic") |

Table 2. Risk of bias assessment of the studies based on The Modified Newcastle–Ottawa tool for cross-sectional studies

|  | Comparability | | | | | Outcome: | | Total score |
| --- | --- | --- | --- | --- | --- | --- | --- | --- |
|  | **1- Representativeness of the sample** | **2. Sample size** | **3. Non-respondents** | **4. Ascertainment of the exposure (risk factor):** | **5. Comparability of subjects in different outcome groups on the basis of design or analysis. Confounding factors controlled.** | **6. Assessment of outcome:** | **7. Statistical test** |  |
| Xu et al. (2025) | 0 | 0 | 1 | 2 | 0 | 1 | 1 | 5 |
| Toska et al. (2025) | 1 | 1 | 0 | 2 | 1 | 1 | 1 | 7 |
| Stankovic and Slavkovic (2025) | 1 | 1 | 1 | 2 | 1 | 1 | 1 | 8 |
| Rinaldi etal. (2025) | 1 | 0 | 0 | 2 | 1 | 1 | 1 | 6 |
| Othman et al. (2025) | 1 | 1 | 0 | 2 | 0 | 1 | 1 | 6 |
| Moisoglou et al. (2025a) | 1 | 1 | 0 | 2 | 1 | 1 | 1 | 7 |
| Moisoglou et al. (2025b) | 1 | 1 | 0 | 2 | 1 | 1 | 1 | 7 |
| Moisoglou et al. (2025c) | 1 | 1 | 0 | 2 | 1 | 1 | 1 | 7 |
| Kang et al. (2025) | 0 | 1 | 1 | 2 | 1 | 1 | 1 | 7 |
| Gün et al (2025 a) | 1 | 0 | 0 | 2 | 0 | 1 | 1 | 5 |
| Gün et al. (2025 b) | 1 | 0 | 0 | 2 | 1 | 1 | 1 | 6 |
| Galanis et al (2025 a) | 0 | 1 | 0 | 2 | 1 | 1 | 1 | 6 |
| Galanis et al. (2025 b) | 1 | 1 | 0 | 2 | 1 | 1 | 1 | 7 |
| Fathulaela et al. (2025) | 0 | 1 | 0 | 2 | 0 | 1 | 1 | 5 |
| Ardıç and Erişen (2025) | 0 | 1 | 0 | 2 | 1 | 1 | 1 | 6 |
| Moisoglou et al. (2024a) | 1 | 0 | 0 | 2 | 1 | 1 | 1 | 6 |
| Moisoglou et al. (2025d) | 0 | 1 | 0 | 2 | 1 | 1 | 1 | 6 |
| Galanis et al. (2024a) | 0 | 1 | 1 | 2 | 1 | 1 | 1 | 7 |
| Galanis et al. (2024b) | 0 | 0 | 0 | 2 | 1 | 1 | 1 | 5 |
| Galanis et al. (2024c) | 0 | 1 | 1 | 2 | 1 | 1 | 1 | 7 |
| Galanis et al. (2024d) | 0 | 1 | 1 | 2 | 1 | 1 | 1 | 7 |
| Ventura-Silva et al. (2026) | 1 | 0 | 1 | 2 | 1 | 1 | 1 | 7 |
| Moisoglou et al. (2026) | 1 | 1 | 0 | 2 | 1 | 1 | 1 | 7 |
| Ghahramani et al.(2026) | 1 | 1 | 0 | 2 | 1 | 1 | 1 | 7 |
| Guberti et al. (2026) | 1 | 1 | 0 | 2 | 1 | 1 | 1 | 7 |
| El-Sayed et al. (2026) | 1 | 1 | 0 | 2 | 1 | 1 | 1 | 7 |
| Belli and Erçelik (2026) | 1 | 1 | 0 | 2 | 0 | 1 | 1 | 6 |
| Bilgiç et al. (2026) | 1 | 1 | 0 | 2 | 1 | 1 | 1 | 7 |
| Danacı et al. (2025) | 1 | 1 | 1 | 2 | 0 | 1 | 1 | 7 |
| Boy et al. (2026) | 1 | 1 | 0 | 2 | 1 | 1 | 1 | 7 |
| Hu et al. (2025 | 0 | 1 | 1 | 2 | 1 | 1 | 1 | 7 |
